# Perfusion estimation from dynamic non-contrast computed tomography using self-supervised learning and a physics-inspired U-net transformer architecture

**DOI:** 10.1007/s11548-025-03323-2

**Published:** 2025-01-20

**Authors:** Yi-Kuan Liu, Jorge Cisneros, Girish Nair, Craig Stevens, Richard Castillo, Yevgeniy Vinogradskiy, Edward Castillo

**Affiliations:** 1 Department of Biomedical Engineering, The University of Texas at Austin, Austin, TX USA; 2 Division of Pulmonary and Critical Care, Beaumont Health, Royal Oak, MI USA; 3 Division of Radiation Oncology, Beaumont Health, Royal Oak, MI USA; 4 Division of Radiation Oncology, Emory University, Atlanta, GA USA; 5 Division of Radiation Oncology, Thomas Jefferson University, Philadelphia, PA USA

**Keywords:** Pulmonary perfusion, Computed tomography, Self-supervised learning, Vision transformer

## Abstract

**Purpose:**

Pulmonary perfusion imaging is a key lung health indicator with clinical utility as a diagnostic and treatment planning tool. However, current nuclear medicine modalities face challenges like low spatial resolution and long acquisition times which limit clinical utility to non-emergency settings and often placing extra financial burden on the patient. This study introduces a novel deep learning approach to predict perfusion imaging from non-contrast inhale and exhale computed tomography scans (IE-CT).

**Methods:**

We developed a U-Net Transformer architecture modified for Siamese IE-CT inputs, integrating insights from physical models and utilizing a self-supervised learning strategy tailored for lung function prediction. We aggregated 523 IE-CT images from nine different 4DCT imaging datasets for self-supervised training, aiming to learn a low-dimensional IE-CT feature space by reconstructing image volumes from random data augmentations. Supervised training for perfusion prediction used this feature space and transfer learning on a cohort of 44 patients who had both IE-CT and single-photon emission CT (SPECT/CT) perfusion scans.

**Results:**

Testing with random bootstrapping, we estimated the mean and standard deviation of the spatial Spearman correlation between our predictions and the ground truth (SPECT perfusion) to be 0.742 ± 0.037, with a mean median correlation of 0.792 ± 0.036. These results represent a new state-of-the-art accuracy for predicting perfusion imaging from non-contrast CT.

**Conclusion:**

Our approach combines low-dimensional feature representations of both inhale and exhale images into a deep learning model, aligning with previous physical modeling methods for characterizing perfusion from IE-CT. This likely contributes to the high spatial correlation with ground truth. With further development, our method could provide faster and more accurate lung function imaging, potentially expanding its clinical applications beyond what is currently possible with nuclear medicine.

## Introduction

Pulmonary functional imaging (PFI), including ventilation and perfusion, is a key component of many clinical applications, such as identifying pulmonary abnormalities like pulmonary embolism, pulmonary hypertension, chronic obstructive pulmonary disease (COPD), and thrombotic sequelae in COVID-19 patients.^10,13,29^ PFI additionally has been used to guide functional avoidance radiotherapy.^14,15,17,36^ Currently, PFI is primarily acquired via nuclear medicine, including single-photon emission CT (SPECT/CT),^14,28^ positron emission tomography (PET),^25^ or through MRI with hyperpolarized gas^27^ and contrast-enhanced MRI.^12^ Among these methods, SPECT/CT is the most common, but requires ionizing radiation and produces low-resolution perfusion images. On the other hand, PET/CT provides higher quality images, but requires more radiation dose and a longer scanning time.^18,23^ While MRI does not require radiation, the availability of hyperpolarized gas and expensive equipment effectively limit its clinical applicability. Contrast-enhanced MRI/CT methods require injections of contrast agents that are contraindicated in some patients.^31^ Therefore, an increasing amount of studies have been proposed for deriving PFIs from non-contrast CT imaging studies.^2^

CT-derived functional imaging methods can be classified into two types: physics-based numerical methods and deep learning-based (DL) methods. The physics-based methods began with pulmonary ventilation and the work of Simon et al*.*, which estimated ventilation using the differences between registered Hounsfield units (HU) within inhale and exhale CT image pairs(IE-CT).^32^ More recently, the integrated formulation of the Jacobian (IJF) method for estimating ventilation incorporated robustness by solving a constrained linear least squares problem to recover pulmonary volume changes.^4^ This work addressed numerical instability associated with prior methods^5^ and was extended to derive a surrogate for pulmonary perfusion, namely regional mass differences between spatially corresponding inhale and exhale volumes.^6^ Although these numerical methods can provide a strong rationale and rigorous derivation, they are still susceptible to the effects of image noise, artifacts, and potential errors in their required image processing pipelines. Specifically, physics-based numerical methods require image segmentation and deformable image registration (DIR) of IE-CTs prior to the ventilation/perfusion calculations. As such, they are heavily reliant on the DIR and segmentation results.^38^

Recently, DL approaches have undergone rapid development and demonstrated superiority in image processing applications.^2^ Machine learning models have been proposed for clinical applications that involve four-dimensional CT (4DCT) or inhale/exhale CT image pairs (IE-CT), including prediction of PFIs.^26,30,31^ The current state-of-the-art DL-based model achieved 0.7 Spearman’s correlation and shows great potential for generating pulmonary perfusion images from CT imaging.^30^ However, due to the limited availability of large medical image databases for training, current models typically apply supervised learning to small datasets, which may lead to overfitting and poor generalizability to diverse datasets. As such, many existing DL methods are limited to task-specific applications.^1,9,21,26,33,38^

As a subset of self-supervised learning, self-supervised pre-training methods overcome the issues of limited labeled data by learning representative features from larger unlabeled datasets. Several self-supervised methods have been developed for lung image registration,^34^ tumor characterization,^37^ and pattern recognition.^3^ Motivated by these studies, we aim to apply and validate a deep learning model to predict spatial pulmonary perfusion images from IE-CT. To do this, we adopt a self-supervised learning approach designed to extract implicit features of IE-CT using a larger 4DCT image database collected from multiple sources so that the model may be more generalizable for future clinical applications. We then employ transfer learning to fine-tune our model on a limited number of paired IE-CT and SPECT-perfusion (SPECT-P) images to predict pulmonary perfusion. Through self-supervised learning, we reduce the high-dimensional IE-CT into a lower-dimensional latent feature space, then generate corresponding functional lung images after training with a transfer learning framework. To the best of our knowledge, this is the first study that applies self-supervised deep learning to volumetric pulmonary functional imaging.

## Materials and methods

### Model design overview

**Fig. 1:**
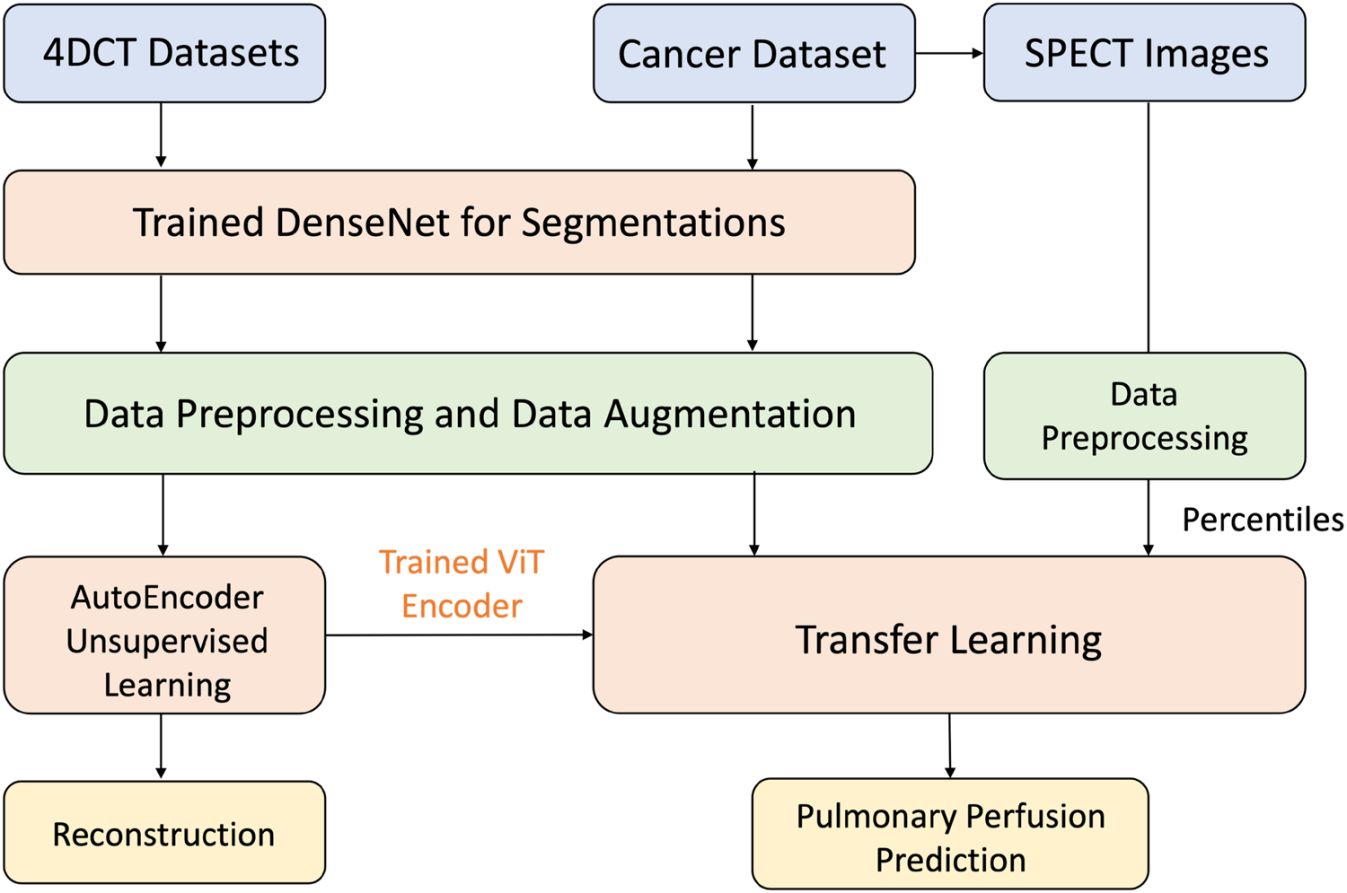
A two-step training framework proposed in this study: a self-supervised learning model is trained on the augmentation removal and reconstruction task of IE-CT scans, using a aggregated 4DCT dataset from multiple different sources. A transfer learning model is then trained for predicting pulmonary perfusion, using the pretrained vision-transformer (ViT) based feature encoder and paired IE-CT and SPECT-P images

The most intuitive deep learning approach for predicting pulmonary perfusion from IE-CT is to train a supervised model with a large number of paired IE-CT and SPECT-P images, but such datasets are not publicly available, limiting model performance and generalizability.^2^ Consequently, previous studies have relied on smaller datasets, which restricts the developed model’s accuracy and applicability. To address this, we propose a self-supervised learning strategy to determine generic meaningful features of the image space, motivated by physics-based methods that require both inhale and exhale CT images to estimate perfusion from pulmonary blood mass distribution changes.^6^ We employ a Siamese Network strategy for a two-channel deep learning model that takes both inhale and exhale images as input for predicting SPECT-P images.^24^ Given the limited paired training data, we propose a two-step training process: first, using self-supervised learning to learn a low-dimensional representation for IE-CT images by reconstructing them from random augmented versions,^22^ and second, applying transfer learning on a smaller dataset of 44 paired IE-CT and SPECTP images to train the perfusion prediction model, utilizing the feature extractor from the self-supervised learning task. See Fig. 1.

### Self-supervised learning stage

**Fig. 2:**
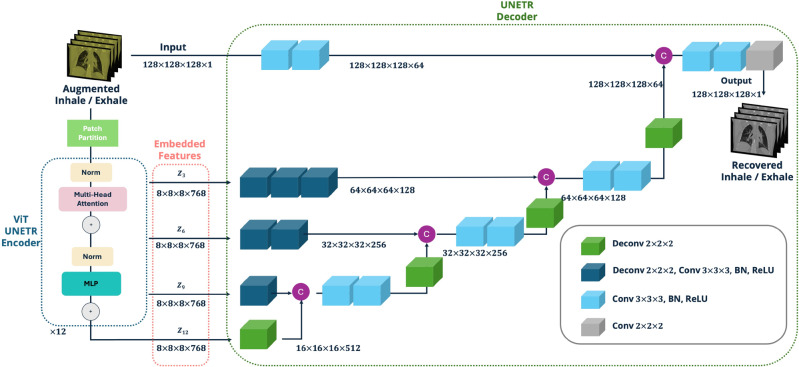
An original UNETR model was applied for the self-supervised learning task: recovering the original image from the randomly augmented inhale or exhale image. The trained UNETR encoder, ViT, was then used for extracting general embedding features from CT images to help transfer learning

IE-CT images include various tissues, such as bones, muscles, cartilages, and other organs. Key features from these images relevant to functional image calculation are lung shape, HU-estimated tissue densities, and volume changes between different respiratory phases.^4,6,32^ However, there is unrelated information, like image noise and background, outside the pulmonary cavity. Thus, to predict perfusion, it is crucial to extract useful information from IE-CT scans and discard insignificant features. Reducing data dimensionality helps avoid the” curse of dimensionality” and distill a robust set of semantic features. In this study, we applied self-supervised learning for dimensionality reduction using the UNETR model, obtaining a robust encoder to distill latent features from many unlabeled IE-CT images.^16^ We adopted an original UNETR model to transform IE-CT images into a representative latent space by capturing essential features from both inhale and exhale phases simultaneously.^11^ This model uses transformer blocks to extract and propagate features into UNETR decoder blocks at different resolutions, ultimately rebuilding the images and learning semantic features such as lung volume changes and HU variations (Fig. 2).

### Training data for self-supervised learning stage

For self-supervised learning, a total of 523 IE-CT images were collected from 9 different 4DCT datasets, including the 3 public datasets 4D-Lung,^20^ DIR-Lab,^7^ and LBCC.^35^ For each image, the maximum inhale phase and maximum exhale phases were used for a IE-CT pair. Among the jointed cohort, 129 cases were acquired for suspected pulmonary embolism patients (Clinical Trials Registration: NCT03183063), while the remaining were acquired for non-small cell lung cancer patients prior to radiotherapy (Clinical Trials Registration: NCT02528942). A DenseNet^19^ pre-trained on the publicly-available COPDGene®^19^ dataset, which contains 7485 breath-hold IE-CT images, was used for left and right lung volume segmentation. The DenseNet is capable of generating stable and reliable 3D lung segmentation that is minimally affected by artifacts from IE-CTs. All the images were pre-processed before the self-supervised learning.

The image pre-processing pipeline applied 523 IE-CT images is described as follows: (1) With lung volume segmentation provided by the pre-trained DenseNet, we separate each IE-CT into left and right lungs, which effectively increases the dataset size by a factor of two, (2) in order to enhance the model’s perceptive efficiency, center cropping is applied with a margin of 5 voxels around the segmentation masks, (3) background volumes (everything outside lung masks) are removed to further focus on pulmonary information, (4) each IE-CT image is down-sampled to a resolution of 128 × 128 × 128 voxels to reduce computational cost and allow for the images to fit within the memory of our available graphics processing units (GPUs) (5) HU values in the images are normalized into densities by the approach first proposed by Simon et al*.* in the context of computing lung compliance.^32^ After pre-processing, a total of 2092 CT images, including inhale and exhale lung of both sides, are generated from 523 cases.

**Fig. 3:**
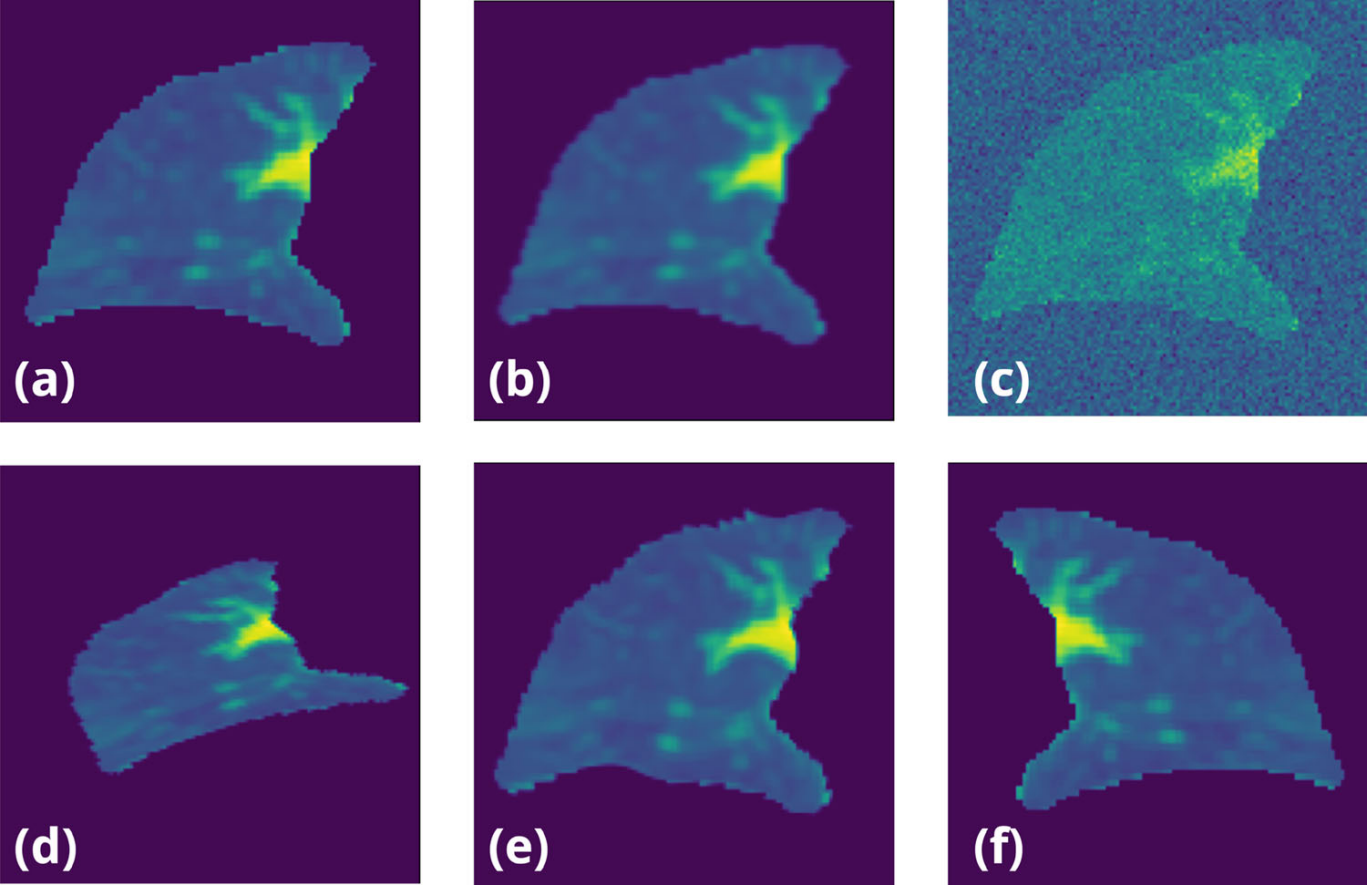
To increase the robustness of models, **a** the origin input image for training were randomly transformed with the following transformations: **b** randomly blurring, **c** adding random noise, **d** random affine transformation, **e** elastic transformation with a random range, and **e** random flipping

To promote the utility of the latent information extracted from the IE-CTs, we increase the difficulty of the autoencoder-based reconstruction task by applying random image augmentations on every IE-CT image pair during training, including randomly blurring, random noising, random affine transformation, elastic transformation with a random range, and random flipping (Fig. 3). Therefore, in order to correctly reconstruct the original images with features extracted from augmented ones, the encoder model must extract features that encode patient-specific information from within the IE-CTs. After self-supervised training, the trained ViT is ready to be applied as a feature extractor for warm-starting training in the transfer learning stage.

### Transfer learning stage

**Fig. 4:**
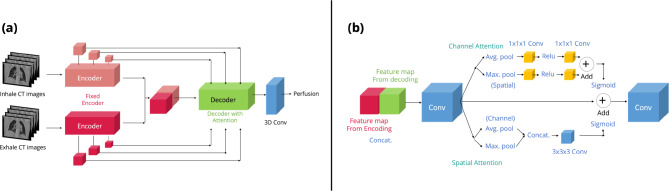
**a** An UNETR-based decoder was trained with features extracted from the paired inhale and exhale CTs by the pre-trained ViT encoder. **b** The attention structure applied in this model, including channel-wised and spatial-wised attention

The self-supervised training provides a robust encoder that transforms IE-CT into a lower-dimensional latent feature space that allow for a straight-forward transfer learning strategy to train the final perfusion prediction model. To do this, the regression decoder trained in the self-supervised learning model is replaced with a modified UNETR decoder. The model weights for the trained encoder are taken as warm-start states to and are fine-tuned during the supervised training process. With a two-channel Siamese strategy,^24^ the inhale and exhale features generated from the same pretrained ViT are concatenated and then propagated into a self-attention block, which includes both spatial and channel attention to further determine useful features for the transfer-learning task^39^ (Fig. 4).

### Training data for transfer learning stage

For the transfer learning stage, we employ data acquired as part of our previous studies on non-small cell lung cancer (clinicalTrials.gov Identifier NCT02528942).^36^ Our dataset contains paired non-contrast 4DCT scans and SPECT-P images for 44 patients, acquired prior to radiotherapy. We utilized the peak inhale and exhale phases from the 4DCT to form the IE-CT pairs as model input. Each CT volume followed the DICOM standard format (512 × 512 pixels per 2D slice image, voxel dimensions of 1.27 mm × 1.27 mm × 3 mm). As in the self-supervised learning pipeline, the same DenseNet pretrained on COPDGene®^19^ was applied for lung volume segmentation of the cancer patient cohort. In order to apply the pretrained ViT encoder on 4DCT images, the same pre-processing pipeline as before was performed here (see Sect. 2.3).

**Fig. 5:**
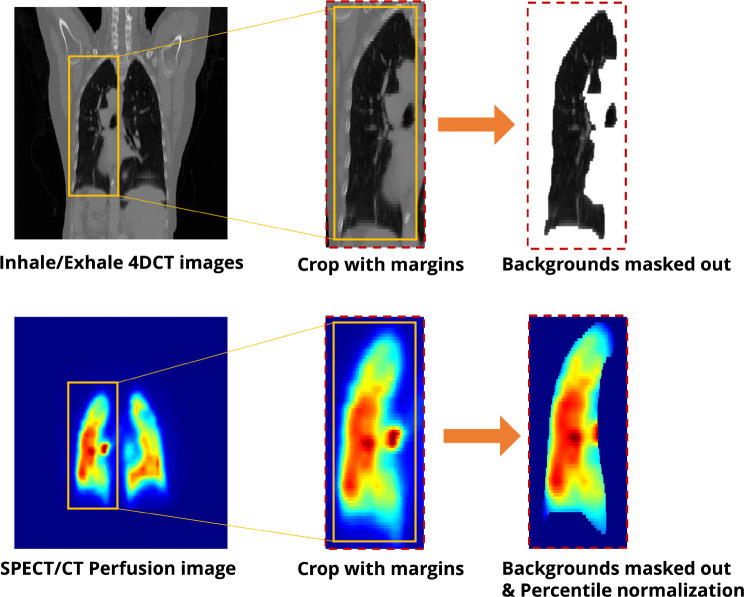
By computing segmentation, all CT images were separated into left and right lungs and cropped with margins. The backgrounds were removed to simplify the data. Similarly, the SPECT/CT images were also pre-processed with the same approaches, but the values were transformed to percentiles for normalization

The SPECT-P images were registered to the 4DCT coordinates using standard affine registration applied to the attenuation correction CT. Then, Steps 1–4 of the pre-processing pipeline 2.3 for CT images were also applied to the corresponding SPECT-P attenuation CT to determine the left/right lung volumes. Different from the normalization step of the CT images, the SPECT-P images were converted into percentile images based on the photon counts (see Fig. 5), in order to handle the wide distribution of intensities common in SPECT-P images. Finally, a moving average with a 3 × 3 × 3 kernel size was applied to smooth the SPECT-P distribution and reduce the effect of artifacts in SPECT images, as done in previous studies^8^.

### Training details and experiments

For all training processes, we randomly separated the dataset into training, validation, and testing sets with 70%, 15% and 15% ratio. The validation sets were used to stop the training process early when the lowest validation loss had not been updated for 150 epochs. As mentioned above (see Sect. 2.3), to make the model more robust, all images were randomly augmented with transforms before training (Fig. 3). The data augmentation prevents over-fitting of the models and make it possible to reuse the lung cancer dataset in the different tasks during both self-supervised and supervised learning stages. The augmentation efficiently addresses the potential data leakage issues caused by the implicit relationship between left and right lung by changing both the contours and the intensities. The Adam optimizer with 10^−3^ learning rate in PyTorch was applied. All processes were executed on a machine with 4 NVIDIA A100 GPUs, each with 80 GB memory storage. We used batch sizes of 12 and applied different loss functions for the training of each phase. For both the augmentation removal task and the perfusion image reconstruction task, we applied mean absolute error (MAE) as objective to generate more general results. For perfusion prediction, lung segmentations are passed to the loss function to define lung volumes, since perfusion is only defined within the lung. Bootstrap sampling was applied 10 times to randomly divide the samples into training, validation, and testing sets.

**Fig. 6:**
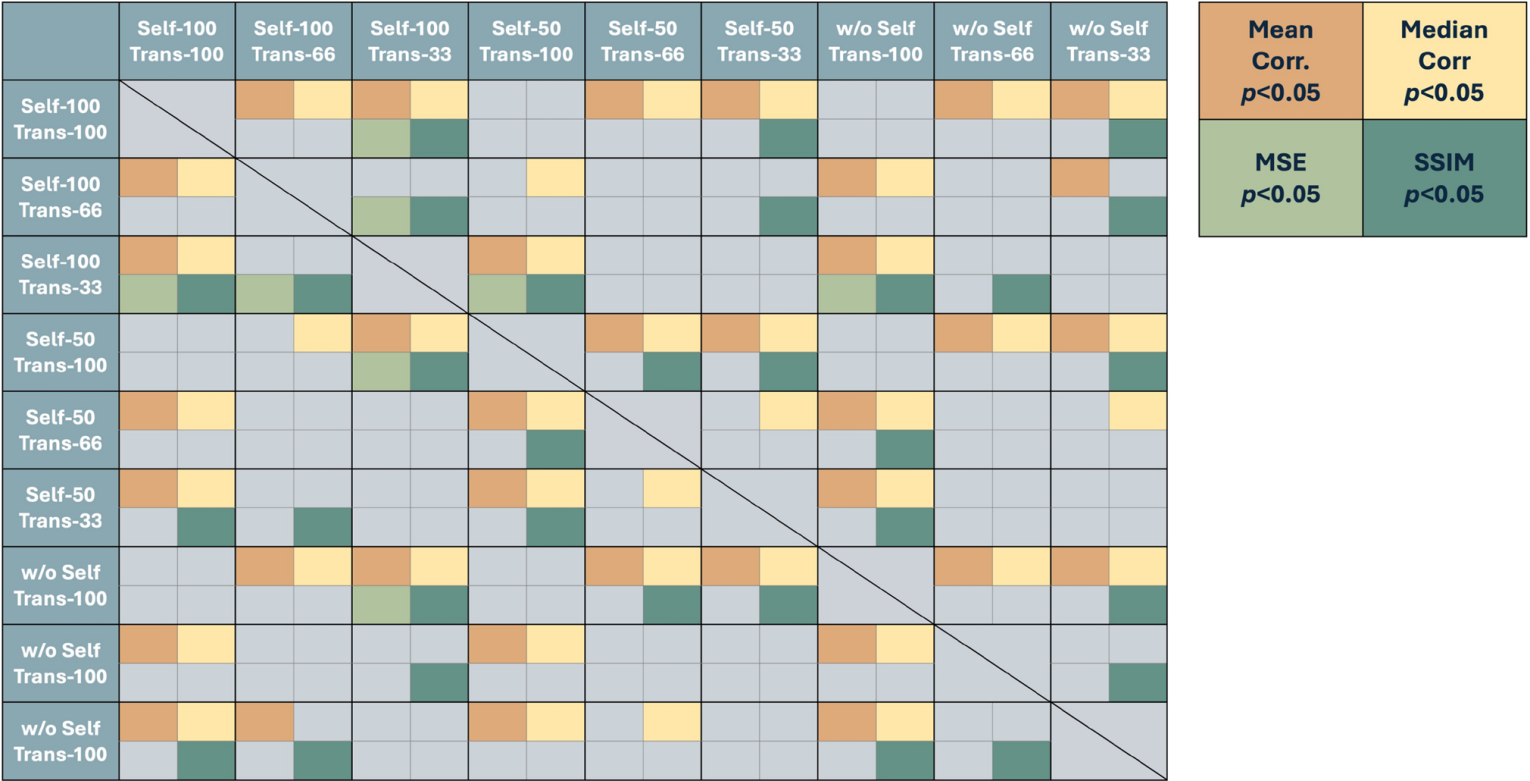
Heatmap illustrating pairwise comparisons of performance metrics across different combinations of self-supervised and transfer learning proportions. Significant differences (*p* < 0.05) are marked using distinct colors for each metric: mean Spearman’s correlation (orange), median Spearman’s correlation (yellow), mean square error (MSE; light green), and volumetric structural similarity index (SSIM; blue-green)

To further investigate the impact of the training data quantity to the perfusion prediction, we compared conditions with different amounts of training data, randomly selected from the full training data set, for both training stages. Specifically, for the self-supervised learning stage, the data used for training were randomly selected from the divided training set with 3 different portions: 100%, 50%, and without self-supervised learning stage. For the transfer learning stage, 3 different proportions were similarly set for randomly selecting the training data: 100%, 66%, and 33%. Thus, we compared the model performance in 9 different combinations that consider both training stages. For each combination, we measured the model performance with four common metrics: mean and median volumetric Spearman’s correlation, mean square error (MSE), and volumetric structural similarity (SSIM). Results from each combinations were compared with paired t-test using fixed data partitions, random seeds, and hyper-parameters for each trial Fig. 6.

## Results

**Fig. 7:**
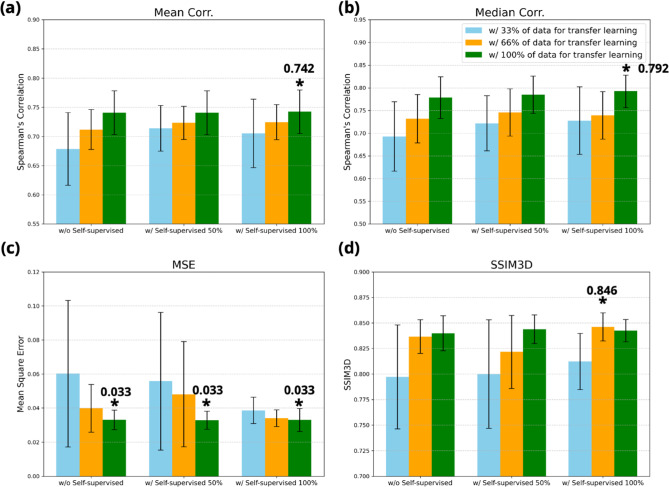
Testing results from 10 different bootstrapping trials that compared prediction and SPECT ground truth in for metrics: **a **the mean Spearman’s correlation, **b** the median Spearman’s correlation, **c** mean square error (MSE), and **d** the volumetric structural similarity. The best performance conditions are marked with *

The average and standard deviation of the four quantitative metrics are shown in Table 1 and Fig. 7. The model’s Spearman’s correlation performances under different conditions varied from 0.678 to 0.742, while the median Spearman correlation varied from 0.693 to 0.792. MSE varied from 0.033 to 0.060 and volumetric SSIM varied from 0.797 to 0.846. Notably, the combination of 100% self-supervised learning and 100% transfer learning achieves the best mean (0.742 ± 0.037) and median (0.792 ± 0.036) Spearman’s correlations, as well as the lowest MSE (0.033 ± 0.0067), alongside a competitive SSIM score (0.842 ± 0.011). These results demonstrate the superiority of leveraging the full dataset under both learning paradigms, especially the transfer learning stage. Although increasing the proportion of transfer learning generally enhances performance, no significant difference was found between results using all the transfer learning data (See Fig. 6). Overall, the results indicate the models can accurately predict the spatial distribution of pulmonary perfusion within lung volumes, especially when incorporating the self-supervised pre-trained feature encoder. The low standard deviations among the bootstrapping showed that our model is stable and likely generalizable, which is a critical quality for any future clinical studies based on the perfusion predictions.

**Table 1:** The mean and median Spearman’s correlations, mean square error (MSE), and volumetric structural similarity (SSIM) from 10 bootstrapping testing data with different amount of training data in self-supervised learning and transfer learning conditions

Self-supervised Learning	Transfer Learning	Mean Corr	Median Corr	MSE	SSIM
0%	33%	0.678 ± 0*.*062	0.693 ± 0*.*076	0.060 ± 0*.*0430	0.797 ± 0*.*051
0%	66%	0.714 ± 0*.*034	0.731 ± 0*.*053	0.040 ± 0*.*0134	0.837 ± 0*.*017
0%	100%	0.741 ± 0*.*037	0.778 ± 0*.*046	**0.033** ± 0*.*0057	0.840 ± 0*.*017
50%	33%	0.714 ± 0*.*039	0.722 ± 0*.*061	0.056 ± 0*.*0404	0.800 ± 0*.*053
50%	66%	0.723 ± 0*.*028	0.746 ± 0*.*052	0.048 ± 0*.*0310	0.822 ± 0*.*036
50%	100%	0.741 ± 0*.*037	0.785 ± 0*.*041	**0.033** ± 0*.*0052	0.844 ± 0*.*014
100%	33%	0.705 ± 0*.*059	0.728 ± 0*.*074	0.040 ± 0*.*0077	0.812 ± 0*.*027
100%	66%	0.724 ± 0*.*030	0.739 ± 0*.*052	0.034 ± 0*.*0048	**0.846** ± 0*.*014
100%	100%	**0.742** ± 0*.*037	**0.792** ± 0*.*036	**0.033** ± 0*.*0067	0.842 ± 0*.*011

The bold values indicate best performance for each metric

**Fig. 8:**
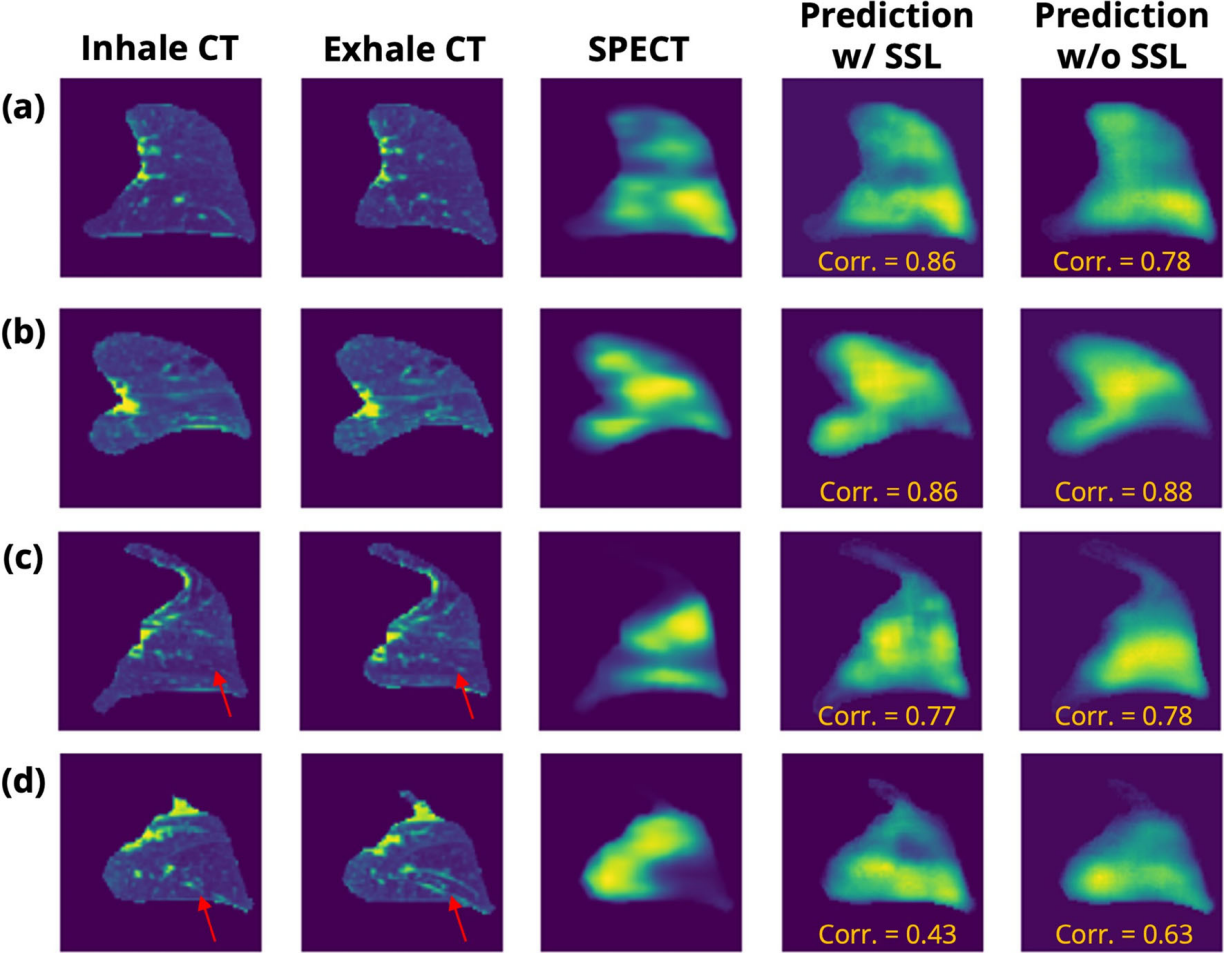
Four qualitative results with inhale and exhale CT images, the corresponding SPECT-P image, and the predicted SPECT-P image from the trained model with all or without self-supervised learning (SSL) stage before transfer learning. Examples with high to low correlation are shown from (**a**) to (**d**). The red arrows show the possible image artifacts from the IE-CTs

Qualitative evaluation of examples with different spatial correlations (see Fig. 8) gives some sense of the fidelity of our model’s prediction. The testing result with a high correlation between the predicted perfusion image and ground truth is shown in Fig. 8a and b, demonstrating the capability of correctly predicting distribution of pulmonary perfusion, including normal pulmonary function and dysfunction. Remarkably, as illustrated in Fig. 8a, the model can accurately predict the normal lung perfusion near the diaphragm, and successfully detect detailed perfusion abnormalities and defects in the middle lung. However, there are also several perfusion images that could not be predicted well, such as Fig. 8c and d, possibly due to the 4DCT artifacts as pointed out with red arrows in Fig. 8 or extreme abnormal lung conditions, such as severe lung fibrosis.

## Discussion

In this study, we successfully developed a deep learning model for predicting 3D pulmonary perfusion images based on non-contrast IE-CTs. To the best of our knowledge, the performance of this study is the current state-of-the-art compared to previous work in the literature, including the physics-based numerical models^6^ or competing DL models.^30^ In terms of spatial Spearman’s correlation, the average testing results outperformed previous models, with a 22% increase (from 0.57 to 0.792) over the established physical model,^6^ and 9.8% increase (from 0.70 to 0.798) over previous state-of-the-art deep-learning models.^30^ The promising performance shows the model’s potential for future clinical applications as a support tool, allowing radiologist to examine predicted pulmonary perfusion images from non-contrast IE-CT. Within the context of lung cancer radiotherapy, IE-CT data is acquired as standard-of-care, meaning that the predicted perfusion information would be available without disruption to the clinical workflow and without the added cost to the patient. Moreover, this framework can also be applied to ventilation imaging^4^ or positron emission tomography (PET).^25^

There are key components that explain the successful performance of our pipeline. First, the self-supervised learning from a larger dataset with 2 thousand images (including left/right and inhale/exhale) helps the model determine generic and useful latent features from the CT images, where the model encoder is trained to reduce the data’s dimensionality while maintaining useful information, allowing the optimization for image regression to robustly converge. Second, this is the first study that applies both inhale and exhale volumetric CT images simultaneously in a ViT with a Siamese framework to predict pulmonary functional images. As pointed out in the previous established numerical method,^6^ perfusion can be modeled as the mass change between different breathing phases. Thus, our Siamese modeling approach is consistent with the known physics behind perfusion estimation from non-contrast CT. Third, the UNETR architecture applies a ViT that processes images as sequential patches and extracts multi-scale meaningful features, like lung volumes, lobe contours, local defects, or vessels. Therefore, the ViT may find more useful information for representing the CT images. Lastly, we also applied spatial-wise and channel-wise self-attention blocks to determine the relationship between the features extracted from inhale and exhale images. Other components of this study, like careful data pre-processing, data augmentation, and lung-volume-focused optimization, are likely contributing factors to the model’s high performance.

However, the difference between the models with and without self-supervised learning before transfer learning was not as large as we expected. One potential explanation for this could stem from the fact that the cases in the lung cancer dataset commonly present (1) lung tumors of various sizes, and in some cases, 2) contain 4DCT artifacts of various severities. It might be that the feature encoder could not generate correct features given these anomalies. However, future ablation studies are needed to further address this question, primarily training and testing on a dataset from a larger cohort of participants with limited 4DCT artifacts. Although there is no statistically significant difference between results obtained with different data amounts in self-supervised learning, the models with self-supervised learning generally performed better and with smaller standard deviation (See Table 1 and Fig. 7) which indicates the pretrained ViT provides a good warm start for extracting features from IE-CTs. Moreover, this approach allows for a much larger unlabeled dataset to be incorporated into model training, thereby mitigating any potential overfitting or lack of generalizability associated with training on a limited number of labeled data.

From the qualitative results showed in Fig. 8, we notice that most of the perfusion distributions are well predicted with medium–high correlation. However, some details in the ground-truth images are not always captured, especially in areas with extremely high photon counts. This could be explained as the model did not learn how to predict localized hot spots, potentially due to the abnormal lung morphology such as severe pulmonary fibrosis, tumors, incomplete lungs after resection, or imaging artifacts from 4DCT or SPECT-P acquisitions (Fig. 8c and d). Further studies are needed to confirm this, particularly applying a 4DCT artifact-correction pipeline to IE-CT images or training on a larger curated dataset with SPECT-P ground-truth.

Although our model demonstrates promising results, there are some limitations in this study. First, the current approach requires lung volume segmentation for removing the background, cropping the lungs, and aiding the optimization. A poorly trained segmentation model could be problematic for our pipeline. Additionally, the quality of registration between SPECT-P scans and IECTs might lead to potential errors when generating the training labels. Thus, further studies are required for accessing how sensitive this approach is to segmentation and registration. Moreover, the developed model requires both inhale and exhale CT scans as inputs. Although 4DCT is standard-of-care for thoracic radiotherapy, IE-CT is not commonly acquired outside of imaging trials, such as COPDGene®. The IE-CT images extracted from 4DCT phases usually contain variable artifacts, making our trained model possibly limited and difficult to apply on unseen breath-hold IE-CT scans. Moreover, the current model has been only trained on non-small cell lung cancer data. Thus, the trained model might not generalize to datasets with different pathologies. Lastly, the deficiencies of SPECT-P, namely, the lower spatial resolution and possible artifacts, will be learned by the model as well. However, our training approach is generalizable and suitable for limited data. Thus, future work is to apply the model to more robust perfusion imaging, such as dual energy CT perfusion and MRI-based perfusion.

## Conclusions

This study is the first to apply self-supervised learning for predicting SPECT pulmonary perfusion using non-contrast 4DCT images. The model achieved state-of-the-art benchmarks, with an average spatial correlation of 0.742 ± 0.037 and 0.792 ± 0.036 for the median within the whole lung volume. Two-step self-supervised learning with the UNETR and Siamese Network allows the model to take both inhale and exhale CT images as input, making our model consistent with known physics governing pulmonary perfusion estimation from non-contrast CT. Our model has the potential to be useful for accelerating clinical processes and supporting disease diagnoses without the need for a nuclear medicine clinic.

## References

[1] Astley JR, Biancardi AM, Marshall H, Hughes PJC, Collier GJ, Hatton MQ, Wild JM, Tahir BA (2023) A hybrid model- and deep learning-based framework for functional lung image synthesis from multi-inflation CT and hyperpolarized gas MRI. Med Phys 50(9):5657–5670. 10.1002/mp.16369 36932692 10.1002/mp.16369 PMC10946819

[2] Astley JR, Wild JM, Tahir BA (2022) Deep learning in structural and functional lung image analysis. British J Radiol 95(1132):20201107 10.1259/bjr.20201107 PMC9153705 33877878

[3] Bajc M, Neilly JB, Miniati M, Schuemichen C, Meignan M, Jonson B (2009) EANM guidelines for ventilation/perfusion scintigraphy: Part 1. Pulmonary imaging with ventilation/perfusion single photon emission tomography. Eur J Nuclear Med Molecular Imag 36(8):1356–1370 10.1007/s00259-009-1170-5 19562336

[4] Castillo E, Castillo R, Vinogradskiy Y, Dougherty M, Solis D, Myziuk N, Thompson A, Guerra R, Nair G, Guerrero T (2019) Robust CT ventilation from the integral formulation of the Jacobian. Med Phys 46(5):2115–2125. 10.1002/mp.13453 30779353 10.1002/mp.13453 PMC6510605

[5] Castillo E, Castillo R, Vinogradskiy Y, Guerrero T (2017) The numerical stability of transformation-based CT ventilation. Int J Comput Assist Radiol Surg 12(4):569–580 28058533 10.1007/s11548-016-1509-x PMC5362676

[6] Castillo E, Nair G, Turner-Lawrence D, Myziuk N, Emerson S, Al-Katib S, Westergaard S, Castillo R, Vinogradskiy Y, Quinn T, Guerrero T, Stevens C (2021) Quantifying pulmonary perfusion from noncontrast computed tomography. Med Phys 48(4):1804–1814. 10.1002/mp.14792 33608933 10.1002/mp.14792 PMC8252085

[7] Castillo R, Castillo E, Guerra R, Johnson VE, McPhail T, Garg AK, Guerrero T (2009) A framework for evaluation of deformable image registration spatial accuracy using large landmark point sets. Phys Med Biol 54(7):1849–1870 19265208 10.1088/0031-9155/54/7/001

[8] Cazoulat G, Balter JM, Matuszak MM, Jolly S, Owen D, Brock KK (2021) Mapping lung ventilation through stress maps derived from biomechanical models of the lung. Med Phys 48(2):715–723 33617034 10.1002/mp.14643 PMC8444226

[9] Chartrand G, Cheng PM, Vorontsov E, Drozdzal M, Turcotte S, Pal CJ, Kadoury S, Tang A (2017) Deep learning: A primer for radiologists. RadioGraphics. 37(7):2113–2131 29131760 10.1148/rg.2017170077

[10] Dhawan RT, Gopalan D, Howard L, Vicente A, Park M, Manalan K, Wallner I, Marsden P, Dave S, Branley H, Russell G, Dharmarajah N, Kon OM (2021) Beyond the clot: perfusion imaging of the pulmonary vasculature after COVID-19. The Lancet Respir Med 9(1):107–116 33217366 10.1016/S2213-2600(20)30407-0 PMC7833494

[11] Dosovitskiy A, Beyer L, Kolesnikov A, Weissenborn D, Zhai X, Unterthiner T, Dehghani M, Minderer M, Heigold G, Gelly S, Uszkoreit J, Houlsby N (2021) An image is worth 16x16 words: Transformers for image recognition at scale. arXiv:2010.11929 [cs].

[12] Eichinger M, Puderbach M, Fink C, Gahr J, Ley S, Plathow C, Tuengerthal S, Zuna I, Müller F-M, Kauczor H-U (2006) Contrast-enhanced 3D MRI of lung perfusion in children with cystic fibrosis—initial results. Eur Radiol 16(10):2147–2152 16673092 10.1007/s00330-006-0257-7

[13] Elojeimy S, Cruite I, Bowen S, Zeng J, Vesselle H (2016) Overview of the novel and improved pulmonary ventilation-perfusion imaging applications in the Era of SPECT/CT. Am J Roentgenol 207(6):1307–1315 27726408 10.2214/AJR.15.15071

[14] Eslick EM, Stevens MJ, Bailey DL (2019) SPECT V/Q in Lung Cancer Radiotherapy Planning. Semin Nucl Med 49(1):31–36 30545514 10.1053/j.semnuclmed.2018.10.009

[15] Faught AM, Miyasaka Y, Kadoya N, Castillo R, Castillo E, Vinogradskiy Y, Yamamoto T (2017) Evaluating the toxicity reduction with computed tomographic ventilation functional avoidance radiation therapy. Int J Radiation Oncol Biol Phys 99(2):325–333 10.1016/j.ijrobp.2017.04.024 PMC5605907 28871982

[16] Hatamizadeh A, Tang Y, Nath V, Yang D, Myronenko A, Landman B, Roth HR, Xu D (2022) UNETR: Transformers for 3D medical image segmentation. In: 2022 IEEE/CVF winter conference on applications of computer vision (WACV), pp 1748–1758, Waikoloa, HI, USA, January 2022. IEEE.

[17] Hoover DA, Capaldi DPI, Sheikh K, Palma DA, Rodrigues GB, Rashid Dar A, Yu E, Dingle B, Landis M, Kocha W, Sanatani M, Vincent M, Younus J, Kuruvilla S, Gaede S, Parraga G, Yaremko BP (2014) Functional lung avoidance for individualized radiotherapy (FLAIR): study protocol for a randomized, double-blind clinical trial. BMC Cancer 14(1):934 25496482 10.1186/1471-2407-14-934 PMC4364501

[18] Huang B, Law MW-M, Khong P-L (2009) Whole-body PET/CT scanning: estimation of radiation dose and cancer risk. Radiology 251(1):166–174 19251940 10.1148/radiol.2511081300

[19] Huang G, Liu Z, van der Maaten L, Weinberger KQ (2017) Densely connected convolutional networks. 4700–4708.

[20] Hugo GD, Weiss E, Sleeman WC, Balik S, Keall PJ, Lu J, Williamson JF (2016) Data from 4D lung imaging of NSCLC patients.

[21] Kajikawa T, Kadoya N, Maehara Y, Miura H, Katsuta Y, Nagasawa S, Suzuki G, Yamazaki H, Tamaki N, Yamada K (2022) A deep learning method for translating 3DCT to SPECT ventilation imaging: First comparison with 81mKr-gas SPECT ventilation imaging. Med Phys 49(7):4353–4364. 10.1002/mp.15697 35510535 10.1002/mp.15697 PMC9545310

[22] Karras T, Aittala M, Hellsten J, Laine S, Lehtinen J, Aila T (2020) Training generative adversarial networks with limited data. Adv Neural Inform Process Syst 33:12104–12114

[23] Kaushik A, Jaimini A, Tripathi M, D’Souza M, Sharma R, Mondal A, Mishra AK, Dwarakanath BS (2015) Estimation of radiation dose to patients from 18FDG whole body PET/CT investigations using dynamic PET scan protocol. Indian J Med Res 142(6):721–731 26831421 10.4103/0971-5916.174563 PMC4774069

[24] Koch G, Zemel R, Salakhutdinov R, et al. (2015) Siamese neural networks for one-shot image recognition. In: ICML deep learning workshop, volume 2, pages 1–30. Lille, Issue: 1.

[25] Le Roux P-Y, Hicks RJ, Siva S, Hofman MS (2019) PET/CT lung ventilation and perfusion scanning using galligas and gallium-68-MAA. Semin Nucl Med 49(1):71–81 30545520 10.1053/j.semnuclmed.2018.10.013

[26] Liu Z, Miao J, Huang P, Wang W, Wang X, Zhai Y, Wang J, Zhou Z, Bi N, Tian Y, Dai J (2020) A deep learning method for producing ventilation images from 4DCT: First comparison with technegas SPECT ventilation. Med Phys 47(3):1249–1257. 10.1002/mp.14004 31883382 10.1002/mp.14004

[27] Mathew L, Wheatley A, Castillo R, Castillo E, Rodrigues G, Guerrero T, Parraga G (2012) Hyperpolarized 3He magnetic resonance imaging: Comparison with four-dimensional X-ray computed tomography imaging in lung cancer. Acad Radiol 19(12):1546–1553 22999648 10.1016/j.acra.2012.08.007

[28] Matuszak MM, Matrosic C, Jarema D, McShan DL, Stenmark MH, Owen D, Jolly S, Kong F-M, Ten Haken RK (2016) Priority-driven plan optimization in locally advanced lung patients based on perfusion SPECT imaging. Adv Radiation Oncol 1(4):281–289 10.1016/j.adro.2016.10.007 PMC5514230 28740898

[29] Mistry NN, Pollaro J, Song J, De Lin M, Johnson GA (2008) Pulmonary perfusion imaging in the rodent lung using dynamic contrast-enhanced MRI. Magn Resonance Med 59(2):289–297. 10.1002/mrm.21353 10.1002/mrm.21353 PMC2738602 18228577

[30] Porter EM, Myziuk NK, Quinn TJ, Lozano D, Peterson AB, Quach DM, Siddiqui ZA, Guerrero TM (2021) Synthetic pulmonary perfusion images from 4DCT for functional avoidance using deep learning. Phys Med Biol 66(17):175005 10.1088/1361-6560/ac16ec 34293726

[31] Ren G, Zhang J, Li T, Xiao H, Cheung LY, Ho WY, Qin J, Cai J (2021) Deep learning-based computed tomography perfusion mapping (DL-CTPM) for pulmonary CT-to-perfusion translation. Int J Radiation Oncol Biol Phys 110(5):1508–1518 10.1016/j.ijrobp.2021.02.032 33689853

[32] Simon BA (2000) Non-invasive imaging of regional lung function using X-Ray computed tomography. J Clin Monit Comput 16(5):433–442 12580227 10.1023/a:1011444826908

[33] Soffer S, Klang E, Shimon O, Barash Y, Cahan N, Greenspana H, Konen E (2021) Deep learning for pulmonary embolism detection on computed tomography pulmonary angiogram: a systematic review and meta-analysis. Scientif Rep 11(1):15814 10.1038/s41598-021-95249-3 PMC8338977 34349191

[34] Tapson VF (2008) Acute pulmonary embolism. New England J Med 358(10):1037–1052. 10.1056/NEJMra072753 18322285 10.1056/NEJMra072753

[35] Vandemeulebroucke J, Rit S, Kybic J, Clarysse P, Sarrut D (2011) Spatiotemporal motion estimation for respiratory-correlated imaging of the lungs. Med Phys 38(1):166–178 21361185 10.1118/1.3523619

[36] Vinogradskiy Y, Castillo R, Castillo E, Schubert L, Jones BL, Faught A, Gaspar LE, Kwak J, Bowles DW, Waxweiler T, Dougherty JM, Gao D, Stevens C, Miften M, Kavanagh B, Grills I, Rusthoven CG, Guerrero T (2022) Results of a multi-institutional phase 2 clinical trial for 4DCT-ventilation functional avoidance thoracic radiation therapy. Int J Radiat Oncol Biol Phys. 112(4):986–995 34767934 10.1016/j.ijrobp.2021.10.147 PMC8863640

[37] Wood KE (2002) Major pulmonary embolism: review of a pathophysiologic approach to the golden hour of hemodynamically significant pulmonary embolism. Chest 121(3):877–905 11888976 10.1378/chest.121.3.877

[38] Yamamoto T, Kabus S, Klinder T, von Berg J, Lorenz C, Loo BW Jr, Keall PJ (2011) Four-dimensional computed tomography pulmonary ventilation images vary with deformable image registration algorithms and metrics. Med Phys. 38(3):1348–1358. 10.1118/1.3547719 21520845 10.1118/1.3547719

[39] Zhang H, Goodfellow I, Metaxas D, Odena A (2019) Self-attention generative adversarial networks. In: Chaudhuri K, Salakhutdinov R,(Eds), Proceedings of the 36th international conference on machine learning, vol. 97 of proceedings of machine learning research, pp 7354–7363. PMLR.

